# Youth’s sense of belonging and associated risk and promotive factors: An ecological systems network analysis

**DOI:** 10.1177/13591045251380305

**Published:** 2025-09-18

**Authors:** Fatima Wasif, Jackson A Smith, Dillon T Browne

**Affiliations:** 1 Department of Psychology, 8430 University of Waterloo, Waterloo, ON, Canada; 2 Centre for Mental Health Research and Treatment, 8430 University of Waterloo, Waterloo, ON, Canada

**Keywords:** Belonging, youth, network analysis, well-being, mental health

## Abstract

**Introduction:**

Belonging is a powerful predictor of positive outcomes in youth, including greater well-being. There remains a pressing need to integrate influences across layers of organization within youths’ developmental contexts to further understand how to enhance belonging amongst this demographic. Here, we investigate: (1) “How do risk and promotive factors converge in relation to belonging among youth?” and (2) “Do risk and promotive factors associate differently with belonging between boys and girls?”.

**Methods:**

Responses from a community-based questionnaire were analyzed to establish ecological systems networks of the interrelationships between youths’ social connections, well-being, belonging, and sociodemographic factors (N_girls_ = 477, N_boys_ = 245; M_age_ = 14.2, *SD* = 2.2 years).

**Results:**

Our findings demonstrate the salience of ethnicity-based discrimination experiences in diminished mental health outcomes and lower belonging among boys. Additionally, we show the crucial link between emotional support from teachers and family with higher belonging for youth. **Conclusions:** We discuss the importance of gender-based considerations when targeting belonging promotion and well-being among children and adolescents.

## Plain Language Summary

When youth feel like they belong to their social networks and surroundings, they do better both mentally and physically. Belonging is linked to numerous positive outcomes for children and adolescents, including better mental health. We need to further our understanding of how youths’ social ties and physical contexts work together to influence belonging to know how to best enhance belonging within this demographic. Here, we investigated how risk and promotive factors, including social support from family, friends, and teachers, mental and physical health, and experiences of discrimination all connect to youth belonging. Further, we looked at how the relationships between these factors may differ across boys and girls, to understand if a gender-sensitive approach to belonging promotion is needed. Responses from 739 children and adolescents on a community-based questionnaire were analyzed using network analysis. We found that emotional support played a unique role in promoting belonging across youth, especially when provided by family and teachers. Interestingly, experiences of being discriminated against based on ethnicity were linked to poorer mental health, specifically greater anger, among boys; but not girls. Girls’ social supports (family, friends, teachers) interacted more than boys’, whose sources of support were largely isolated from each other. Our findings showcase the importance of gender-based considerations when targeting belonging promotion and well-being among children and adolescents.

## Introduction

Adolescence is characterized by significant emotional and social change, including increased reliance on peer relationships and community resources (Albert et al., 2013; Bowker & Ramsay, 2018; Brown & Larson, 2009; Heinsch et al., 2020; Sawyer et al., 2012). During this transitory stage of life—bridging childhood and adulthood and typically spanning the ages of 10–19 (Sawyer et al., 2018)—the importance of belonging becomes increasingly pronounced**.** Belonging (i.e., the subjective sense of feeling needed, valued, and congruent with one’s social and physical environments; Hagerty et al., 1992), is linked to numerous benefits for youth development, including greater well-being, higher academic functioning, and lower participation in risk-taking behaviour, especially among disadvantaged youth (Arslan et al., 2020; Brooks et al., 2012; Magson et al., 2015; Pittman & Richmond, 2007; Tian et al., 2015). Conversely, those who feel marginalized by their social communities report worse mental and physical health outcomes (Ge et al., 2017; Heinrich & Gullone, 2006; Qualter et al., 2013).

Given the benefits of connectedness, it is crucial to better understand the correlates of belonging among this demographic. Research centred on youth belonging has typically involved an investigation of social connectedness, with less consideration paid to the influence of environmental factors (Allen et al., 2021). Moreover, an understanding of how to encourage youth belonging has focused chiefly on schools, with limited emphasis on other aspects of community (Brooks et al., 2012; Resnick et al., 1997). Recent work has outlined the need to conceptualize belonging as a dynamic sentiment that emerges from and is supported by an individual’s social and physical environments (Allen et al., 2021). Thus, there is a pressing need to evaluate how various factors in youth’s physical and social contexts and experiences are interrelated and relate to belonging.

Kern et al. (2020) posits that belonging occurs as a function of the systems within which individuals reside. One of the best-known conceptualizations of these “systems” comes from Bronfenbrenner’s (1979) Ecological Systems Theory (EST). According to EST, youth exist at the centre of a series of interdependent physical settings, extending from one directly influenced by the youth (the *microsystem*) to societal and cultural influences (the *macrosystem*) that impact the youth (Bronfenbrenner, 1979). Revisions of EST reframe ecological settings in terms of the social relationships that surround a child (Neal & Neal, 2013). Within the network view of EST, settings are defined in relation to the direct involvement of the child with others; for example, the *microsystem* is noted as a set of people, including the child, that are engaged in interactions with each other (Neal & Neal, 2013). Network EST offers a theoretical framework to examine overlapping *microsystems* in which the child is directly involved. Within this conceptualization, belonging is ripe for investigation through network analysis, a statistical tool that allows for the construction of psychometric models that display how two variables–termed nodes–are related to each other, controlling for the effect of every other variable in the network (Borsboom et al., 2021). It allows one to simultaneously consider multiple social and physical factors, which may further the current understanding of how belonging emerges, and can be facilitated, among youth.

To examine networks for youth, we must first identify known promotive and risk factors related to social and environmental contexts that can act as representative nodes within our networks. Promotive and risk factors for belonging can be arranged within the EST framework (Allen et al., 2018). Perhaps the most crucial of the ecological networks to belonging are the *microsystems* which encompass a youth’s direct interactions with their family, friends, and teachers. Youth who perceive high social support from teachers, peers, and family, also endorse feeling safe and accepted, which are important ingredients for belonging (Allen et al., 2018). Conversely, negativity in social relationships contributes to a sense of low belonging, in tandem with other socioemotional issues. Poor peer connections can perpetuate feelings of social exclusion among young people, contributing to anxiety and depressive symptoms (La Greca & Lai, 2014). Deterioration of family systems, including perceived parental support, also contributes to loneliness in adolescents (Johnson et al., 2001). Lack of teacher support deprives youth of feeling cared for within schools, risking isolation (Allen et al., 2018).

Across youth, the presence of high levels of psychological distress manifested as depressive symptoms, negative affect, and socio-emotional outcomes (e.g., self-esteem) are linked to low belonging (Korpershoek et al., 2020; Shochet et al., 2011). In contrast, positive emotionality and well-being reciprocally promote and are enhanced by belonging, particularly within the family and school *microsystems* (Jose et al., 2012). In terms of environmental factors, financial marginalization is negatively associated with belonging, such that as material resources increase, so does belonging (Allen et al., 2022). Further, school belonging is lower in youth who perceive their surroundings as unsafe (Craggs & Kelly, 2018). Overall, promotive and risk factors for belonging can be categorized across three discrete areas: social support, psychological well-being, and sociodemographic variables, including the perceived safety of the physical environment. The present study evaluates these indicators of belonging concurrently, parsing apart the most essential ingredients of youth belonging.

### Gender-based considerations for belonging

Gender is known to play a role in creating belonging among youth (Allen et al., 2018). Prior work shows that youth who identify as girls report greater school belonging than those who identify as boys, across diverse samples (Goodenow & Grady, 1993; Hughes et al., 2015; Sánchez et al., 2005; Wagle et al., 2021). In addition, how different facets of social support influence school belonging also differs among teenagers. Family involvement at school was more predictive of school belonging for boys. For girls, greater school belonging was predicted by family involvement within the home (Uslu & Gizir, 2017).

Importantly, there is a paucity of research that evaluates gender-based discrepancies in general belonging, and its predictors, across physical contexts. Furthermore, much previous work has centred on examining divergences in social support provision by gender and subsequent impacts on well-being outcomes (Bokhorst et al., 2010; Rueger et al., 2010; Valcke et al., 2022). While social connectedness is part of belonging, belonging itself is an active process that goes beyond perceived interpersonal connections (Baumeister & Leary, 1995). It is important to compare networks specifically between girls and boys to understand if risk and promotive factors for belonging differ among these groups.

Importantly, before proceeding, we should note that understanding belonging in 2SLGBTQIA + youth is essential. Unfortunately, our current sample did not have enough gender non-conforming youth from the 2SLGBTQIA + community (*N* = 17) to explore networks, a computationally demanding approach. Thus, our analyses refer to boys and girls. In our analyses, this includes transgender youth who identify as boys or girls, but not youth with non-binary genders (e.g., genderqueer, gender-fluid, two-spirit). The study authors, along with the region supporting this research initiative, are aware of the mental health and belonging needs in this community and are actively involved in local advocacy for youth who identify as Two-Spirit, Lesbian, Gay, Bisexual, Transgender, Queer or Questioning, Intersex, Asexual (2SLGBTQIA+), through public demonstrations and attending school board council meetings on flag-related issues. Future iterations of the community survey providing the data this work uses plan to increase outreach and engagement with the 2SLGBTQIA + community, who are indispensable holders of truth regarding the processes that help or hinder belonging.

### The present study

The present study examines interrelationships among social and environmental risk and promotive factors for youth belonging. We attempt to answer the following questions: Q1: How do risk and promotive factors converge to in relation to belonging among youth? Q2: How do ecological networks differ across youth who identify as girls compared to those who identify as boys? Network analysis is exploratory in nature. Consequently, we do not have any strong hypothesis about the structure of the networks.

## Methods

### Participants and procedures

The preregistration, code, and associated analysis for the current study are available at pre-registration and analysis code.

Data were obtained from the Youth Impact Survey (YIS) a regional cross-sectional survey conducted by the Children and Youth Planning Table (CYPT) of Waterloo Region, in partnership with UNICEF Canada, launched in 2021 (CYPT, 2021). The YIS was available for completion for five weeks. Participants were enlisted through non-random sampling methods (e.g., through direct emails, presentations to regional partners). The total survey sample comprised 1074 youth (9–18 years) in the Waterloo Region, which is in Southwestern Ontario, Canada. The current analysis was restricted to a subset of the original survey sample, comprising 739 youth, who provided complete responses to relevant variables of interest. This is because, at the time of data analysis, network analysis packages have not been sufficiently developed to incorporate methods of missing data estimation (e.g., full information maximum likelihood, multiple imputation). Little’s MCAR test (Li, 2013) determined that data within the original sample was not missing completely at random. However, follow-up independent samples *t-tests* comparing those who completed survey items compared to those that did not, showed that completers did not differ significantly from non-completers in terms of age, gender, ethnicity, and socioeconomic status. On average, participants in the study sample were 14.2 years old (*SD* = 2.2). Approximately 64.5% of the participants identified as female, 33.2% as male, and 2.3% as non-binary. Most youths in the sample were of European descent (66.7%), while racialized youth formed 33.3% of the sample. Within minoritized youth, 10.3% reported being of South-Asian descent, followed by East-Asian participants (6.1%). Racialized demographics within the study sample largely reflect those of the Region of Waterloo (33.3% compared to 29% recorded on the 2021 Canadian census; Government of Canada, 2023). Complete demographic details are provided in the supplemental materials (Table S1).

### Measures

With support from UNICEF Canada and the Canadian Index of Well-Being at the University of Waterloo, researchers identified existing validated survey items, taken largely from Statistics Canada. Subsequently, these items were reviewed, selected, and approved by youth. They were then mapped onto the multidimensional model of well-being that youth had already co-constructed with facilitators at the CYPT. Consultation between researchers (including the data partner for Waterloo region, Dr. Dillon Browne, C.Psych.) and a review of the literature on youth belonging led to the selection of 29 YIS questionnaire items for the current study, thought to be most relevant as nodes for the intended network analysis for the current study. Items corresponded with five general themes, including social support, psychological well-being, and sociodemographic variables. Complete variable names and descriptions are provided in Table 1. Notably, since item-level network analysis involves singular items represented as nodes, the internal consistency of scales was not relevant. Further, all item responses were provided through Likert scales, unless specified otherwise. Complete item statements and Likert scale details are provided in the supplemental materials (Table S2).

**Table 1.** Description of study variables and network nodes.

Node	Variable name	Description
BELONGING	BELONGING	Sense of belonging
FAMILY (Provide help)	SUPPORT1	Family willingness to provide help
FAMILY (Emo support)	SUPPORT2	Family provision of emotional support
FAMILY (Share problems)	SUPPORT3	Ability to share problems with family
FAMILY (Decision making)	SUPPORT4	Family help with making decisions
FRIEND (Provide help)	SUPPORT5	Friend willingness to provide help
FRIEND (Reliable help)	SUPPORT6	Reliability of friend help during difficult times
FRIEND (Share emo)	SUPPORT7	Ability to share joys and sorrows with friends
FRIEND (Share problems)	SUPPORT8	Ability to share problems with friends
TEACHER (Acceptance)	TEACHER1	Perceived teacher acceptance for self
TEACHER (Care)	TEACHER2	Perceived teacher care for self
TEACHER (Trust)	TEACHER3	Perceived trust in teacher
TEACHER (Interest)	TEACHER5	Perceived teacher interest in self
TEACHER (Friendliness)	TEACHER6	Perceived teacher friendliness toward self
TEACHER (Fairness)	TEACHER8	Perceived teacher fairness
LIFESAT	LIFESAT	Life satisfaction
LIFEWORTH	LIFEWORTH	Feeling that life is worthwhile
SYMPTOM (Depressed)	SYMPTOM4	Low mood or depressed
SYMPTOM (Anger)	SYMPTOM5	Irritability or bad temper
SYMPTOM (Nervousness)	SYMPTOM6	Nervousness
SYMPTOM (Sleep diff)	SYMPTOM7	Sleep difficulties
STRESS (Daily)	DAILYSTRESS	Stress experienced on a day-to-day basis
MNTLHLTH	MNTLHLTH	Self-reported mental health
PHYSHLTH	PHYSHLTH	Self-reported physical health
WELLOFF	WELLOFF	Perceived family wealth
SAFETY	SAFETY	Perceived neighbourhood safety
DISCRIM (Ethnicity)	DISCRIM3	Ethnicity-based discrimination

#### Social connections

Belonging (BELONGING) was assessed by asking youth how they would describe the strength of their sense of belonging to their community.

##### Family support

Four items (SUPPORT 1, 2, 3, 4) from the YIS survey corresponded to youth’s experience of social support from their family (e.g., “My family really tries to help me.”)

##### Friend support

Four items (SUPPORT 5, 6, 7, 8) from the YIS survey corresponded to youth’s experience of social support from their peers and friends (e.g., “My friends really try to help me.”)

##### Teacher support

Six items (TEACHER 1, 2, 3, 5, 6, 8) from the YIS survey corresponded to youth’s attitude toward their teachers’, focusing on the level of social support children and teens derived from this source. Examples include, “I feel that my teachers accept me as I am.”

#### Psychological well-being

Eight items from the YIS survey corresponded to the youth’s well-being as well as their perceived level of psychological distress. Note also that the YIS provided youth with links to national and regional distress lines and counselling services, should they need extra support after answering questions about their feelings and tough experiences.

Hedonic well-being (LIFESAT) involved youth reporting on their current life fulfillment. Eudaimonic well-being (LIFEWORTH) was represented by youths’ feeling that the things they do in life are worthwhile.

Four items (SYMPTOM 4, 5, 6, 7) on the YIS questionnaire were chosen to denote psychological distress, with participants asked to note how often in the last 6 months, they were low, irritable, nervous, or had sleep difficulties. Participants also self-assessed their mental health (MNTLHLTH), as well as the amount of stress (DAILYSTRESS) they experienced in life on most days.

#### Sociodemographic covariates

Six items (WELLOFF, SAFETY, PHYSHLTH, DISCRIM2, DISCRIM3) on the YIS questionnaire were chosen to characterize the individual, demographic, environmental, and social characteristics of participants within our study sample that could relate to belonging and well-being. These included subjective socioeconomic status and perceived neighbourhood security and safety. Physical health was self-assessed. Youth also reported on whether they had been discriminated against because of their gender identity or ethnicity and/or culture in the past year, with responses reported on a categorical scale. Among our sample, no participants selected “Does not apply/Not sure” as a response.

### Analytical plan

The data were analyzed using R, version 4.2.1 (The R project for statistical computing, 2022). Because data included both continuous and categorical variables, we used Mixed Graphical Models (MGMs) to construct our cross-sectional networks. Consequently, the following packages were used in R: *qgraph, psych, bootnet, igraph,* and *ggplot* (Epskamp et al., 2012; Haslbeck & Waldorp, 2020). In total, we had 26 continuous variables and two categorical ones. Item-level network analysis for the full sample involved three steps: (1) estimating and visualizing the networks, (2) evaluating centrality measures for the established networks, and (3) investigating network stability and accuracy.

#### Missing data

The use of MGMs to analyze our dataset variables entailed the listwise deletion of all participants with any missing values for our chosen nodes. Consequently, the current study sample comprised 739 youth.

#### Descriptive statistics and outlier management

Item distribution and normality were estimated through exploring descriptive statistics using the *psych* and *summarytools* packages within R (Comtois, 2022; Revelle, 2023). Univariate outliers were detected using standard box plots for each continuous variable. Twenty-two multivariate outliers were detected post-winsorization, using the Mahalanobis Distance (MD) method. Results discussed within the paper are with unaltered variables since (1) the kurtosis and skew values for all continuous variables within the dataset were within normality limits (−3 to +3 for skewness, −10 to +10 for kurtosis; Kline, 2011) and (2) variable means between datasets that included and excluded multivariate outliers were comparable (*ps* > .05).

#### Network estimation and visualization

To construct the networks, we used the *bootnet* R package (Epskamp et al., 2017). Estimating MGMs requires specifying the type, as well as the number of levels of each variable (Haslbeck et al., 2021). In our dataset, every continuous variable was noted as “g” for type (signifying their Gaussian distribution), while the ethnicity-discrimination item was noted as “c” for categorical. The number of levels for all continuous variables was noted as “1” as per convention. For the categorical variable, the number of levels was indicated to be “2” representing response options, “Yes” and “No”. The strength an edge must be to be displayed within the network was based on the Extended Bayesian Information Criterion (EBIC), with a hyperparameter of γ = 0.25, and estimates combined across nodewise regressions using the OR-rule. Post-estimation, the MGM was visualized using the *qgraph* package (Epskamp et al., 2012). Please note that the gender-discrimination node was dropped from our network models, owing to the it being unconnected to any nodes. The final models retained 27 nodes, with 26 continuous variables, and one categorical one.

#### Network centrality measures

Key centrality metrics were estimated to examine the importance of variables within the network. This included node strength and bridge strength (Opsahl et al., 2010; Robinaugh et al., 2016). Statistical estimates and plots for centrality measures were determined using the *igraph* package (Csardi & Nepusz, 2006).

#### Network stability and accuracy

To ascertain the stability and accuracy of the network, the stability of the edge weights, significance of the edge-weight differences, and node strength differences were evaluated. Edge-weight variation was examined by non-parametric bootstrapping within 95% of the confidence interval, with 1000 iterations (Epskamp et al., 2017). In addition, the stability of the centrality measures was also computed utilizing correlation stability (CS)-coefficients (Epskamp et al., 2017), whereby only those coefficients above 0.25 were interpreted.

#### Evaluating group differences

We evaluated differences amongst the sample group across boys and girls by first conducting independent samples *t*-tests, adjusting for multiple comparisons to reduce the false discovery rate. In addition, we computed the effect size for each variable that significantly differed between groups, using Cohen’s *d* criteria (Goulet-Pelletier & Cousineau, 2018). Comparisons between networks were carried out using the *NetworkComparisonTest* (NCT) package (Van Borkulo et al., 2022). The NCT statistically compares the difference between two networks based on multiple invariance measures (overall network structure, global network strength, and edge strength). Network structure is assessed through the *M* statistic, which represents the maximum difference in edge strength of the two networks, while network strength is assessed through the *S* statistic, which represents the total value of all the edges in a network (Van Borkulo et al., 2022).

## Results

### Descriptive statistics

Descriptive statistics for each item in the networks are presented in Table 2. Kurtosis and skew values for the full sample indicate that normality estimates were met. Trends indicate that youth generally reported receiving high levels of support from family, friends, and teachers, and that their sense of belonging to their community was somewhat strong. At the same time, they indicated experiencing psychological distress between every month to every week on average, with most days being a bit stressful. Mean self-reported mental health was lower than self-indicated physical health, although both were between fair and very good, overall. Mean perception of family wealth within the sample fell between average and quite well-off, and most youth reported feeling reasonably safe in their physical environments. However, a sizeable proportion reported experiences of discrimination based on their ethnic identity (*n* = 75; 10.1%), gender identity (*n* = 39; 5.3%), and both their gender and ethnicity (*n* = 9; 1.2%).

**Table 2.** Full sample descriptive statistics of variables of interest (*N* = 739).

	*M*	*(SD)*	Skew	Kurtosis
BELONGING	2.76	0.75	−0.34	−0.06
SUPPORT1	4.27	0.86	−1.14	1.12
SUPPORT2	3.95	1.05	−0.81	−0.07
SUPPORT3	3.71	1.16	−0.62	−0.51
SUPPORT4	4.28	0.79	−1.24	2.00
SUPPORT5	3.91	0.97	−0.82	0.42
SUPPORT6	3.88	1.06	−0.92	0.33
SUPPORT7	4.09	1.00	−1.20	1.17
SUPPORT8	3.88	1.06	−0.89	0.27
TEACHER1	4.24	0.80	−0.98	0.79
TEACHER2	3.97	0.93	−0.79	0.28
TEACHER3	3.70	1.00	−0.54	−0.30
TEACHER5	3.83	0.95	−0.59	−0.14
TEACHER6	4.31	0.69	−1.06	2.02
TEACHER8	3.97	0.92	−0.94	0.80
LIFESAT	6.64	2.29	−0.69	0.05
LIFEWORTH	6.65	2.46	−0.70	−0.12
PHYSHLTH	3.45	1.06	−0.30	−0.50
MNTLHLTH	2.76	1.19	0.14	−0.86
SYMPTOM4	2.57	1.44	0.40	−1.22
SYMPTOM5	2.68	1.37	0.29	−1.17
SYMPTOM6	2.96	1.47	0.03	−1.40
SYMPTOM7	2.82	1.56	0.19	−1.50
DAILYSTRESS	2.98	0.99	0.15	−0.46
SAFETY	3.43	0.66	−0.87	0.26
WELLOFF	3.62	0.81	0.02	−0.21

Gender-specific estimates are presented in Table 3, along with comparative independent sample *t*-tests showcasing significant differences between girls and boys across variables. Notably, 4.6% of girls indicated that they had been discriminated against because of their gender identity (*n* = 22) and 11.3% reported experiencing discrimination based on their ethnic identity (*n* = 54). Four (0.54%) reported experiencing both gender and ethnicity-based discrimination. Among boys, 2% (*n* = 5) reported gender-based discrimination, 7.3% (*n* = 18) reported ethnicity-based discrimination, and 0.27% (*n* = 2) noted experiencing both forms of prejudice.

**Table 3.** Gender-specific descriptive statistics of variables of interest (Girls *N* = 477; Boys *N* = 245).

	Girls			Boys		
	M	*(SD)*	M	*(SD)*	*p*	Effect Size
BELONGING	2.81	0.75	2.72	0.72		
SUPPORT1	4.24	0.88	4.35	0.80		
SUPPORT2	3.90	1.08	4.07	0.96	*	0.12
SUPPORT3	3.67	1.20	3.86	1.04	*	0.16
SUPPORT4	4.28	0.80	4.33	0.75		
SUPPORT5	3.94	0.96	3.83	0.97		
SUPPORT6	3.91	1.08	3.83	1.02		
SUPPORT7	4.15	0.97	3.96	1.04	*	0.19
SUPPORT8	3.91	1.05	3.82	1.08		
TEACHER1	4.27	0.78	4.22	0.80		
TEACHER2	3.94	0.95	4.04	0.89		
TEACHER3	3.64	1.02	3.86	0.92	**	0.22
TEACHER5	3.85	0.94	3.80	0.96		
TEACHER6	4.30	0.70	4.33	0.69		
TEACHER8	3.96	0.94	4.00	0.88		
LIFESAT	6.54	2.28	7.00	2.20	**	0.20
LIFEWORTH	6.57	2.50	6.96	2.27	*	0.16
PHYSHLTH	3.43	1.08	3.53	0.99		
MNTLHLTH	2.62	1.14	3.12	1.20	***	0.43
SYMPTOM4	2.77	1.44	2.11	1.32	***	0.48
SYMPTOM5	2.88	1.35	2.22	1.28	***	0.49
SYMPTOM6	3.17	1.45	2.45	1.39	***	0.51
SYMPTOM7	2.97	1.55	2.46	1.52	***	0.33
DAILYSTRESS	3.12	0.96	2.66	0.94	***	0.48
SAFETY	3.40	0.65	3.52	0.65	*	0.18
WELLOFF	3.65	0.84	3.60	0.77		

*Note.* Independent samples *t*-tests were performed to examine mean differences between boys and girls for each variable. *p < .05, **p < .01, ***p < .001.

### Pairwise comparisons between groups

Independent sample *t*-tests (see Table 3) indicate that perceptions of belongingness did not differ between groups. However, there were several notable differences between boys and girls with regards to various factors that relate to belongingness. Girls reported having less family support than boys, in terms of their family’s ability to provide emotional help and support (*t* = −2.10, *SE* = 0.08, *p* = .04) and to be there as a sounding board to share problems with (*t* = −2.16, *SE = 0.09, p* = .03). They also noted having less trust in their teachers than boys (*t* = −2.78, *SE* = 0.08, *p* = .006). In contrast, boys indicated being less able to share their joys and sorrows with their friends than girls (*t* = 2.43, *SE =* 0.08*, p* = .02). Furthermore, girls indicated having worse well-being outcomes than boys, noting less life satisfaction (*t* = −2.56, *SE* = 0.18, *p* = .01), lower life worth (*t* = −2.03, *SE* = 0.19*, p* = .04), worse self-reported mental health (*t* = −5.51, *SE* = .09*, p* < .001) and more frequent occurrences of low mood (*t* = 6.00, *SE* = 0.11*, p* < .001), bad temper (*t* = 6.24, *SE* = 0.10*, p* < .001), nervousness (*t* = 6.42, *SE* = 0.11*, p* < .001), and sleep difficulties (*t* = 4.21, *SE = 0.12, p* < .001). While the perception of wealth did not differ significantly between both groups, girls did indicate perceiving their neighbourhood as more unsafe than boys (*t* = −2.35, *SE =* .05*, p* = .02). Effect sizes for group differences ranged from negligible to medium, with the biggest magnitude of difference observed between the frequency of nervousness girls reported compared to boys.

### Network structure

#### Full sample network

**Figure 1.**
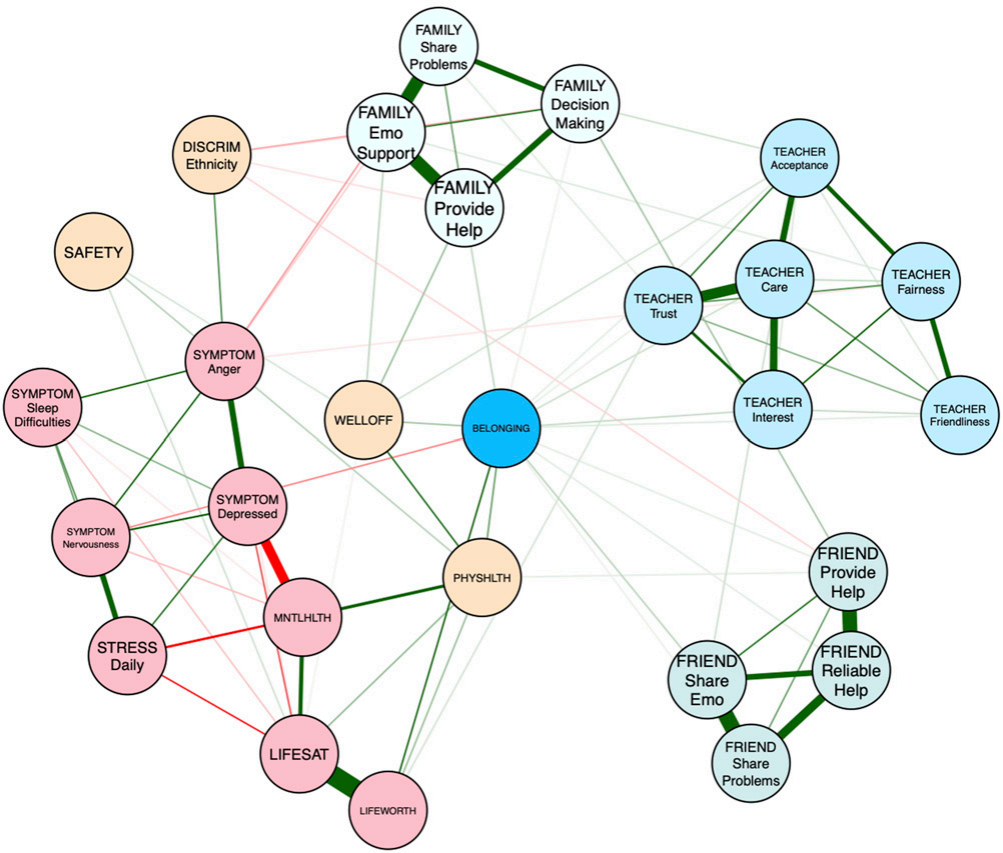
Risk and promotive factors for belonging among youth (*N* = 739). Note. Green-coloured edges denote positive associations, while red edges indicate negative relationships. The thicker the line, the stronger the edge.

Table 1 shows the description of the nodes displayed in all network figures. The network of risk and promotive factors for youth in our community sample is visualized in Figure 1. Out of 351 possible connections, 87 were retained in the full network (*P*_*density*_ = 0.25). Edge weights ranged from −0.32 (*feeling low or depressed–self reported mental health*) to 0.53 (*life satisfaction–life worth)* with a mean edge weight of .02. Within the network, three clusters of highly connected “support” sources arise, denoting family, friends, and teachers. Ethnic discrimination was negatively linked to youths’ perception of family support, particularly in relation to the provision of help, including emotional support and decision-making aid. Furthermore, friend provision of help was also negatively related to ethnicity-targeting discrimination experiences. Ethnic discrimination was also directly related to irritability and bad temper within the network. Family support was related to perceived family wealth, which positively impacted belonging, such that those reporting higher wealth, also noted higher belonging.

#### Gender-specific networks

**Figure 2.**
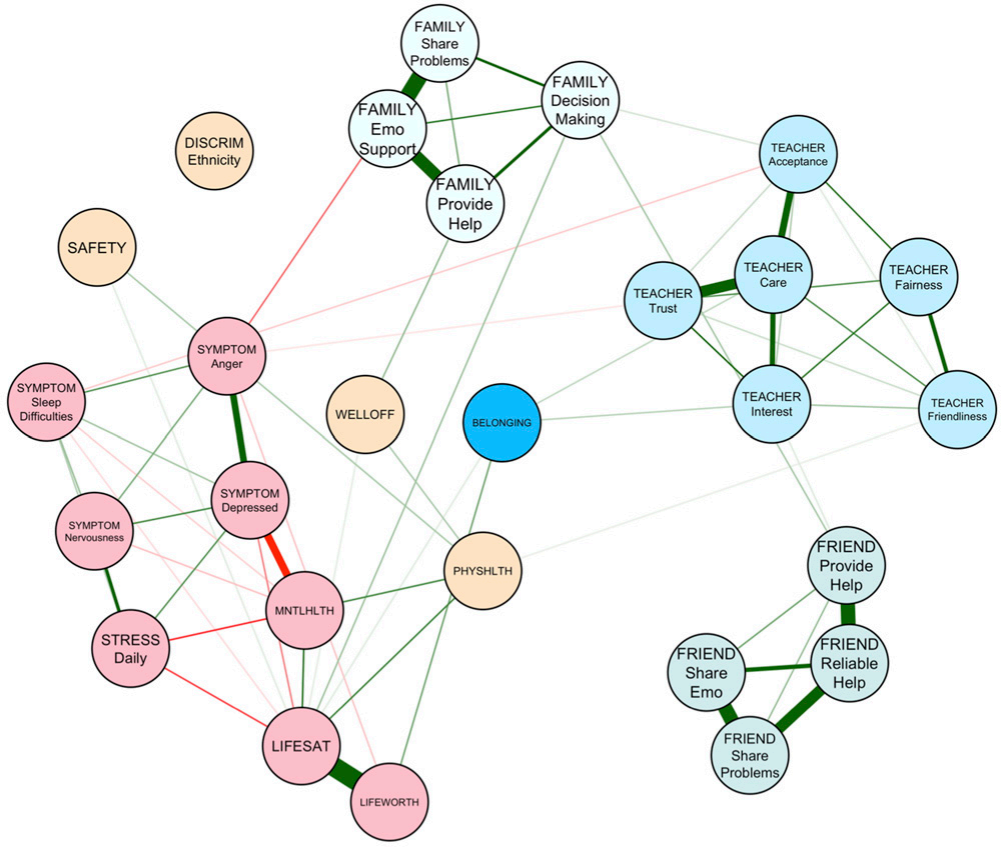
Risk and promotive factors for belonging among girls (*N* = 477).

**Figure 3.**
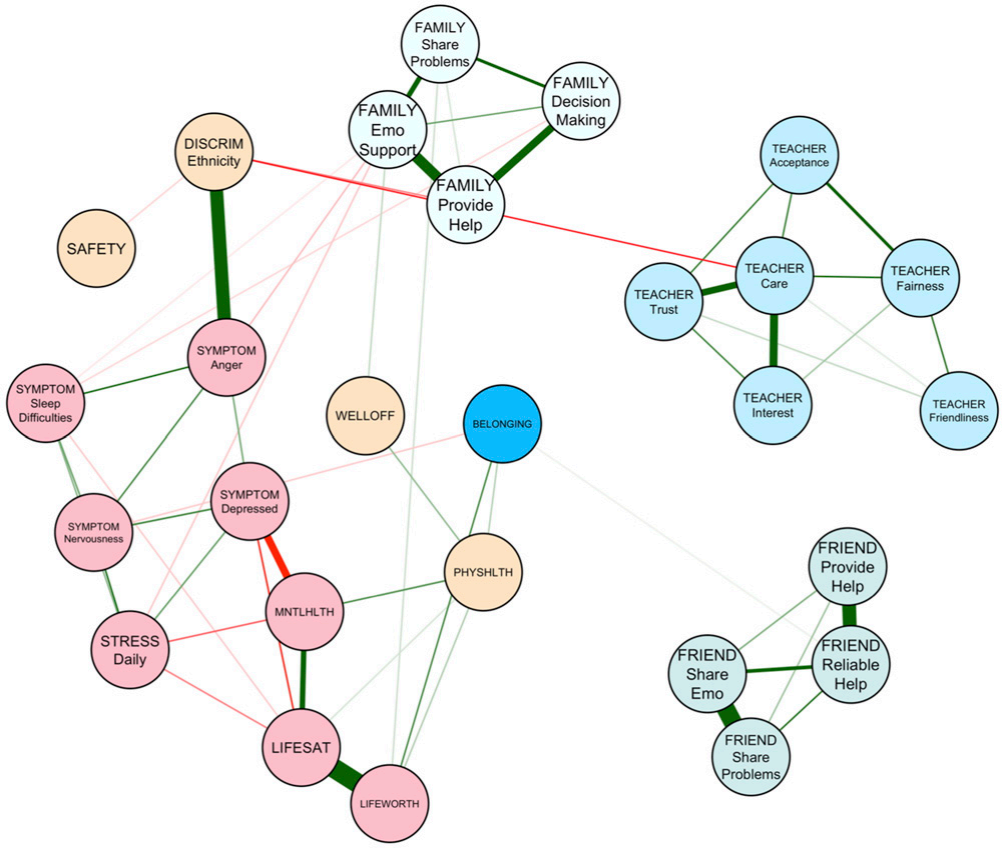
Risk and promotive factors for belonging among boys (*N* = 245).

The networks of risk and promotive factors for girls and boys within our community sample are visualized in Figures 2 and 3. Of 351 possible edges, 64 were retained in the girls’ network (*P*_*density*_ = .18) and 56 were retained in the boys’ network (*P*_*density*_ = .16). Mean edge weight in the girls’ and boys’ networks were .019 and .018, respectively. Edge weights for the girls’ networks ranged from −.29 (*self-reported mental health – feeling low or depressed*) to .52 (*life worth – life satisfaction*). Edge weights for the boys’ networks ranged from −.30 (*self-reported mental health – feeling low or depressed*) to .52 (*life worth – life satisfaction*). Interestingly, while ethnicity-based discrimination was unconnected edges in the girls’ network, ethnicity-based discrimination had several connections in the boys’ network, the strongest of which were with *bad temper* and *irritability* and *experiencing teachers as caring*. Furthermore, belonging was not directly connected to friend support in the girls’ network but was to the reliable provision of friend help in the boys’ network.

### Node centrality

**Figure 4.**
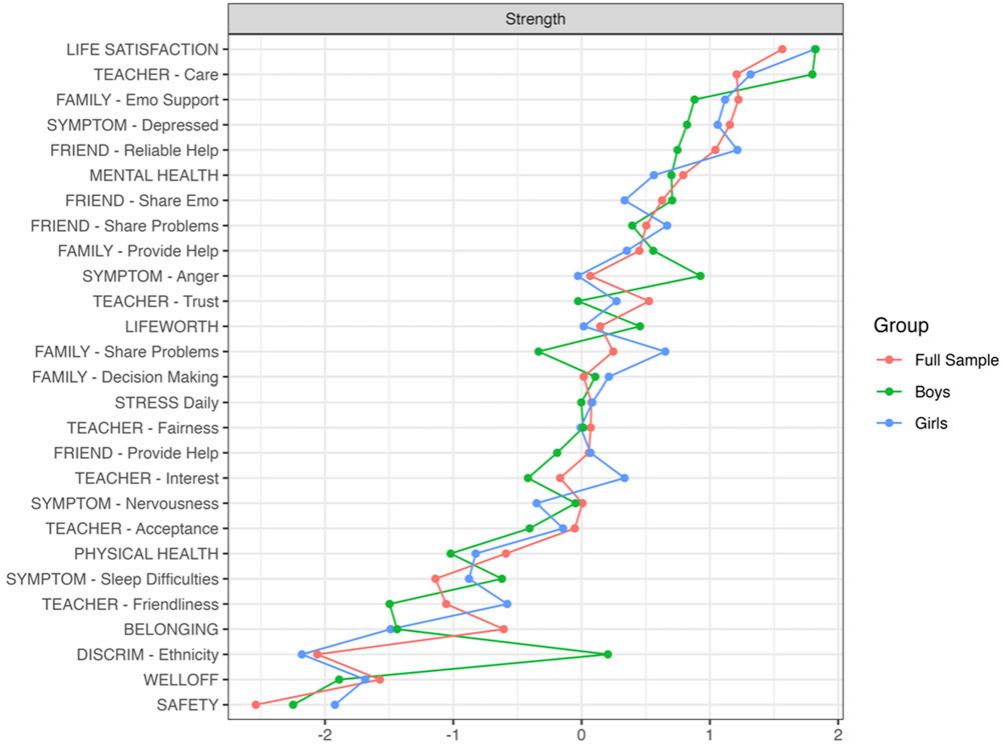
Strength centrality estimates across nodes for all networks (full, girls, and boys). Note. Estimates are standardized across measures for ease of interpretation within the figure.

Figure 4 displays strength centrality estimates. Node centrality was assessed through standardized estimates of node strength (CS coefficient: .75) and bridge strength. Within the full sample, the nodes with highest strength centrality were *life satisfaction* (1.56), *family emotional support* (1.22)*, teacher care* (1.21), *feeling low or depressed* (1.16), and *reliability of friend’s helping during difficult times* (1.04). *Safety* (SAFETY) had the lowest strength estimate with a standardized value of −2.54. When only looking at associations between communities of nodes, the nodes with the highest bridge strength centrality were *mental health* (0.71), *physical health* (0.57), and *belonging* (0.57).

Node centrality in the girls’ network of belonging was assessed through standardized estimates of strength (CS-coefficient: .75). Within the girls’ sample, the nodes with the highest strength centrality were *life satisfaction* (1.82), *teacher care* (1.32), *reliability of friend’s helping during difficult times* (1.21), *family emotional support* (1.12), *feeling low or depressed* (1.06); the least central symptom was *ethnicity-based discrimination* (−2.18). With regards to bridge strength in the girls’ network, *mental health* (0.65), *life satisfaction* (0.51), and *physical health* (0.40) were the most central.

Node centrality of the boys’ network of belonging was also assessed through standardized estimates of strength (CS-coefficient: .44). Within the boys’ sample, the nodes with the highest strength centrality were *life satisfaction* (1.82), *teacher care* (1.80), *irritability or bad temper* (0.93), *family emotional support* (0.88), and *feeling low or depressed* (0.82); the least central symptom was *safety* (−2.25). In the boys’ network, the nodes with the highest bridge strength centrality were *ethnicity-based discrimination* (0.69), *mental health* (0.56), *feeling low or depressed* (0.48), and *irritability or bad temper* (0.45).

### Network stability

Network accuracy was first assessed through edge weight variation, with narrow confidence intervals around estimated edge weights indicative of high stability (Epskamp et al., 2017). Our sample bootstrapped confidence intervals for all three networks were sizeable. While wider confidence intervals indicate a need to consider edge strength differences with caution, they do not preclude the interpretation of the presence of edges within the network. We also examined the stability of differences between edge weights and node strengths for all our network nodes. Edges within our networks were significantly different from each other and, across networks, the node with the most strength, life satisfaction, was significantly larger than many other nodes.

#### Network comparison test

The network comparison test for girls’ and boys’ revealed that the network structures varied, changing across gender groups (*M* = .40, *p* < .01). In contrast, the global strength was invariant (*S* = .22, *p* = .96). The global strength per group was 8.75 (girls) and 8.54 (boys).

Table 4 shows edge labels and significance values obtained from a post-hoc review of edges that differed significantly between boy and girl networks. Several edges were larger in the girls’ network, including *family willingness to offer emotional help* ∼ *youth’ sharing of problems with family* and *irritability* ∼ *low mood*. Relationships between ethnicity-based discrimination and other nodes like family willingness to provide help, teacher care, anger, and safety were present in the boys’ network but not the girls’ network. In contrast, the edge between peer willingness to provide help and perceived teacher interest was present in the girls’ network but not the boys, and there was also a direct connection between family willingness to provide decision-making support and life satisfaction for girls’ that is absent for boys. Relatedly, life worth and sharing problems with family was present in the boys’ network, but not the girls.’

**Table 4.** Edges that differ significantly between the girls’ and boys’ network.

Variable 1	Variable 2	Edge value (Girls)	Edge value (boys)	*p*
FAMILY (Emo support)	FAMILY (Share problems)	0.45	0.26	.02
FRIEND (Reliable help)	FRIEND (Share problems)	0.36	0.17	.04
FRIEND (Provide help)	TEACHER (Interest)	0.02	0.00	.03
TEACHER (Care)	TEACHER (Fairness)	0.00	0.18	.04
FAMILY (Decision making)	LIFESAT	0.07	0.00	.02
FAMILY (Share problems)	LIFEWORTH	0.00	0.04	.04
TEACHER (Trust)	SYMPTOM (Anger)	−0.02	0.00	.03
TEACHER (Acceptance)	SYMPTOM (Sleep difficulties)	−0.03	0.00	.02
SYMPTOM (Depressed)	SYMPTOM (Anger)	0.27	0.10	.04
FAMILY (Provide help)	DISCRIM (Ethnicity)	0.00	−0.09	.05
TEACHER (Care)	DISCRIM (Ethnicity)	0.00	−0.16	.01
SYMPTOM (Anger)	DISCRIM (Ethnicity)	0.00	0.40	.02
SAFETY	DISCRIM (Ethnicity)	0.00	−0.03	.03
FAMILY (Emo support)	DAILYSTRESS	0.00	−0.04	.04

## Discussion

Belonging is a powerful force for youth development and is linked to numerous beneficial outcomes across psychological, social, and academic domains (Allen et al., 2018; Maurizi et al., 2013). Our paper is unique in its examination of the conditional associations between youth belonging and a multitude of risk and promotive factors from an ecological systems framework, allowing us to understand the concurrent influence of social and environmental factors on belonging among youth. Further, our analysis provided novel evidence of how ecological networks differ systematically among boys and girls, highlighting the need for gender-sensitive belonging and well-being promotion within this demographic.

### Social factors

Within our sample, social promotive factors such as family provision of help, sharing problems with family, trust in teachers, teacher care, teacher interest, sharing emotional states with friends, friend provision of help, and the reliability of friend’s help during difficult times were all directly associated with greater belonging among youth. The present findings suggest that overall, belonging is greater in the presence of emotional support, regardless of the source of support. Further, given the strong interrelationships among items within each “cluster” of social support, other items within each cluster influence belonging via nodes with direct links to belonging itself. For example, the presence of emotional support by the youth’s family is linked to the family’s material resources. Youth who reported greater perceived wealth endorsed higher belonging, both directly and through enhanced family provision of help, emotional and otherwise.

The social *microsystems* that interacted most within our sample were family and teachers. Peers maintained a distinct presence in youths’ life, with few interrelationships emerging between friend support nodes and parent or teacher nodes, though family decision-making help did support youths’ perceptions of their friends as being willing to help them, and teacher acceptance was linked to sharing emotional states with friends. This finding somewhat deviates from past work that demonstrates how attachment to family figures heightens perceptions of extrafamilial connections (including peers) as warm and accepting, linking parental support to peer and teacher relationships (Al-Yagon, 2016). It is possible that our networks reflect the role that peer relationships attain as children age into adolescence. The mean age of our sample was approximately 14 years, commensurate with the developmental stage at which youth begin to rely more on friends for assistance than adult figures (Brown & Larson, 2009). Regardless, our work also emphasizes the necessity of utilizing a network view of ecological systems to better understand youth belonging. Clearly, all *microsystems* that a child is a part of do not interact equally with each other (i.e., the *mesosystem*), and yet interrelationships between them exist in a manner that can be mobilized in interventions aimed at promoting belonging.

### Individual factors

At the individual level, promotive factors directly associated with belonging included eudaimonic well-being, physical health, and perceptions of life being worth living. Only one risk factor—nervousness—was directly and negatively associated with belonging. This association is consistent with previous research that has found that interpersonal challenges associated with anxiety, such as difficulties interacting with people, impede high quality relationships among teens (Liu et al., 2024). From an ecological systems perspective, nervousness may hinder a youth’s ability to make effective connections with social agents within the *microsystem*, such as with peers and teachers, which may attenuate youth belonging. Other individual-level risk factors for belonging encompass several symptoms related to mental ill-health, including feeling low or depressed and experiencing anger or irritability (Lester et al., 2013; Parr et al., 2020). In our analysis, these symptoms were indirectly associated with belonging via nervousness, as well as through compromised mental health, lower life satisfaction, and perceptions of life being worthwhile. For example, low mood was related to worse reported mental health, which was associated with lower life satisfaction, which was, in turn, positively linked to how worthwhile youth reported their life to be.

### Environmental factors

One factor that was also notably linked to mental health symptoms, specifically bad temper and irritability, were experiences of ethnic discrimination. Experiences of prejudice are unique in that they traverse social and physical contexts, given that they are most immediately perpetuated by social agents, but have a notable, negative, and harmful impact on the environment an individual occupies. Associations between belonging and well-being have also been observed in other studies, including work with youth who have experienced other forms of discrimination, like prejudice against their status as a sexual minority (Perales & Campbell, 2020). Other aspects of the physical environment, such as perceived neighbourhood safety, were not directly linked to belonging, although subjective family wealth was.

### Gender-based considerations

Within our study sample, levels of belonging were similar among boys and girls. This is contrary to past findings reporting gender-based group differences in belonging, with girls endorsing greater belonging (Neel & Fuligni, 2013). Importantly, much prior work has looked exclusively at belonging within schools, while our study asks youth to rate their belonging in relation to their communities, which expands past the school environment. Additionally, as teens age, girls begin to report less belonging while boys’ belonging remains stable, bringing both groups’ belonging in line with each other (Neel & Fuligni, 2013; Witherspoon & Ennett, 2010).

Despite levels of belongingness being similar for boys and girls, differences in the relative importance of specific risk and promotive factors that influence belonging emerged. While both boys and girls rely on teacher care and family emotional support, relationships between sharing problems with family and family offering emotional support, as well as sharing problems with friends and receiving reliable help from friends, were significantly stronger in the girls’ network compared to the boys’ network. It may be that girls derive greater benefit in terms of consistent emotional support provision from family and friends when sharing their problems with both sources than boys. It is also possible that social support takes a “tough love” approach with boys, emphasizing ideals typical of traditional ideas of masculinity, such as being told to “man up” rather than being offered warmth and sensitivity (APA, 2018). This may make boys less open to being vulnerable with close others, despite evidence suggesting that close, strong relationships with same-and-opposite gender friends enhance male well-being throughout the lifespan (Smiler & Heasley, 2016).

Prejudice again emerged as a dual social-environmental risk factor linked to poorer mental health and belonging. Among boys, the presence of irritability and bad mood emerged in relation to experiences of ethnic discrimination; this association was absent from the girls’ network. Relatedly, boys reported low family willingness to offer emotional help, care from teachers, and feeling less safe when experiencing ethnic discrimination. It is also worth noting that sources of support within the boys’ network act as islands, largely disconnected from each other. Moreover, among boys, the only source of support directly associated with belonging was reliable help from friends. Additionally, family support was only indirectly associated with boy’s sense of belonging through well-being outcomes, and support from teachers was only indirectly associated with belonging through multiple steps between ethnic discrimination and nervousness. For girls, more connections exist between these three facets of support.

Overall, the current study suggests that promoting belonging requires a nuanced, gender-sensitive approach. Specifically, emotional support from peers is valuable in making girls feel valued and accepted, while affective encouragement from teachers and parents is universally beneficial for youth belonging. Furthermore, special attention must be given to boys’ vulnerability to irritability and anger, particularly within the context of discriminatory experiences that enhance isolation by potentially making important facets of the *microsystem*, like family, less available as an avenue of social support.

### Implications for supporting youth well-being

Our findings offer important insights for parents and caregivers, educators, clinicians, and youth-focused organizations. Mental health professionals and school-based practitioners should prioritize strengthening youths’ relationships with adults and peers as key pathways to enhancing belonging and well-being. This may include interventions to support family emotional attunement, encouraging caregivers to express interest and positive regard for their child, and guiding teachers to recognize and affirm students’ strengths and contributions. Strengthening parent-teacher collaboration can further coordinate support across home and school contexts. Additionally, training educators in relational and trauma-informed practices may have broad benefits, particularly for youth experiencing discrimination or emotional distress. Youth-focused organizations also play a critical role in translating research into accessible language to raise public and policy-maker awareness about the links between belonging and well-being. Such efforts can help inform inclusive policies and programming that more effectively promote youth connection, inclusion, and resilience.

### Limitations

Several limitations to the current study should be noted. Our analysis of gender differences in networks was restricted to those who self-identified as girls and boys (including transgender youth). Future research should employ stratified sampling techniques to recruit a greater proportion of gender-diverse youth. This issue is presently being addressed by the study team in follow-up work. Additionally, data collection for the present study occurred during the summer of 2021, during the COVID-19 pandemic. It is possible that the unique conditions that the pandemic created impacted the strength and types of connections we observed in the networks. For example, remote learning disrupted educational experiences for young people, including impeding their access to peer and teacher support networks (Blackwell et al., 2022). Replications of the current study now that schools have largely returned to in-person teaching will be vital to reinforce our understanding of youth networks. Finally, an interesting relationship between experiences of ethnic discrimination and perceiving one’s family as less willing to provide help emerges in the boys’ network. We are unable to establish causal patterns in our analysis given the cross-sectional nature of our data. It will be crucial for prospective longitudinal studies to examine the interplay among these proximal and distal risk and promotive factors that relate to belongingness over time.

## Conclusion

This study demonstrated that both promotive factors within youths’ *microsystems* and intra-individual mental health risks are meaningfully linked to youths’ sense of belonging. Parental and teacher support were positively associated with youth belonging, while nervousness showed a direct negative association, potentially reflecting how internal distress may hinder social connection. Our findings also underscore the importance of a gender-sensitive approach to belonging promotion, with ethnic discrimination emerging as a salient risk factor for irritability, low mood, and diminished belonging among boys. We identified ways in which parents, educators, and communities can foster belonging through emotional support, strengths-based and trauma-informed practice, and the cultivation of inclusive environments. Future research should build on this work by employing longitudinal designs to clarify how specific promotive and risk factors—like ethnic discrimination—influence the developmental interplay between youth mental health, social support, and community belonging over time.

## Supplemental Material

Supplemental Material - Youth’s sense of belonging and associated risk and promotive factors: An ecological systems network analysis Supplemental Material for Youth’s sense of belonging and associated risk and promotive factors: An ecological systems network analysis by Fatima Wasif, Jackson A. Smith, and Dillon T. Browne in Clinical Child Psychology and Psychiatry.

## Data Availability

The data are not publicly available due to restrictions regarding information that could compromise the privacy of the research participants.*
