# Supplementary material for: Youth’s sense of belonging and associated risk and promotive factors: An ecological systems network analysis
Source: Clin Child Psychol Psychiatry. 2025 Sep 18;31(1):172–94. doi: 10.1177/13591045251380305 (PMC12705891; doi:10.1177/13591045251380305)
Supplement: Supplemental Material - Youth’s sense of belonging and associated risk and promotive factors: An ecological systems network analysis [file sj-pdf-1-ccp-10.1177_13591045251380305.pdf]

Tables

Table S1. *Sociodemographic Characteristics of Youth in the Study Sample (N=739)*

	M	*(SD)*
Age	14.2	(2.2)
	n	%
Sex		
Female	495	67.3
Male	241	32.7
Gender		
Girl/Woman	477	64.5
Boy/Man	245	33.2
Non-Binary	17	2.3
Race		
White (European descent)	491	66.7
South Asian (East Indian, Pakistani, Bangladeshi, etc.)	76	10.3
East Asian (Chinese, Korean, Japanese, etc.)	45	6.1
Black (African, Afro-Caribbean, African-Canadian descent etc.)	37	5.0
Middle Eastern (Arab, Persian, West Asian descent, etc.)	31	4.2
Southeast Asian (Filipino, Vietnamese, Cambodian, etc.)	26	3.5
Another Race	37	5.0
Prefer Not to Answer	25	3.4

Note. Percentages may not add to 100% due to rounding.

Table S2. *Item statements and Likert scales*

Variable Name	Item Statement	Likert Scale
BELONGING	"How would you describe your sense of belonging to the community? Would you say it is:"	"Very weak" (1) to "Very strong" (4)
SUPPORT1	"My family really tries to help me"	"Strongly disagree" (1) to "Strongly agree" (5)
SUPPORT2	"I get the emotional help and support I need from my family"	
SUPPORT3	"I can talk about my problems with my family"	
SUPPORT4	"My family is willing to help me make decisions"	
SUPPORT5	"My friends really try to help me"	"Strongly disagree" (1) to "Strongly agree" (5)
SUPPORT6	"I can count on my friends when things go wrong"	
SUPPORT7	"I have friends with whom I can share my joys and sorrows"	
SUPPORT8	"I can talk about my problems with my friends"	
TEACHER1	"I feel that my teachers accept me as I am,"	"Strongly disagree" (1) to "Strongly agree" (5)
TEACHER2	"I feel that my teachers care about me as a person"	
TEACHER3	"I feel a lot of trust in my teachers"	
TEACHER5	"My teachers are interested in me as a student"	
TEACHER6	"Most of my teachers are friendly"	
TEACHER8	"Our teachers treat us fairly"	
LIFESAT	Satisfaction with life in general (Hedonic wellbeing based on OECD 11-point scale)	"Very dissatisfied" to "Very satisfied"
LIFEWORTH	Feeling that things done in life are worthwhile (EUDAIMONIC wellbeing based on OECD 11-point scale)	"Not at all" to "Completely"
SYMPTOM4	"In the last 6 months, how often have you felt *felt low (depressed)"*	"Rarely or never" (1) to "About every day" (5)
SYMPTOM5	"In the last 6 months, how often have you *had irritability or bad temper"*	
SYMPTOM6	"In the last 6 months, how often have you *felt nervous"*	
SYMPTOM7	"In the last 6 months, how often have you *had difficulties in getting to sleep"*	
DAILYSTRESS	"The amount of stress I experience in life, most days…"	"Extremely stressful" (1) to "Not at all stressful" (5); reverse coded
MNTLHLTH	Self-assessed mental health	"Poor" (1) to "Excellent" (5).
PHYSHLTH	Self-assessed physical health	

WELLOFF	"How well off do you think your family is?"	"Not at all well off" (1) to "Very well off" (5)
SAFETY	"In general, how safe child or youth feels from crime in their neighbourhood"	"Very unsafe" (1) to "Very safe" (4)
DISCRIM3	"In past year, experienced discrimination because of your *ethnicity or culture"*	(1 = "Yes", 2 = "No", and 3 = "Does not apply/Not sure")

Table S3. *Complete centrality indices across all networks (full, girls, boys).*

**Node**	**Strength**		
	Full Sample	Girls	Boys
MENTAL HEALTH	0.79	0.56	0.70
SAFETY	-2.54	-1.92	-2.25
DISCRIM - Ethnicity	-2.06	-2.18	0.21
SYMPTOM - Depressed	1.16	1.06	0.82
STRESS Daily	0.08	0.08	0.00
WELLOFF	-1.57	-1.69	-1.89
BELONGING	-0.61	-1.49	-1.44
SYMPTOM - Sleep Difficulties	-1.14	-0.88	-0.62
TEACHER - Friendliness	-1.06	-0.58	-1.50
PHYSICAL HEALTH	-0.59	-0.83	-1.02
SYMPTOM - Nervousness	0.01	-0.35	-0.05
SYMPTOM - Anger	0.07	-0.03	0.93
LIFE SATISFACTION	1.56	1.82	1.82
TEACHER - Acceptance	-0.05	-0.14	-0.41
FAMILY - Share Problems	0.25	0.65	-0.34
TEACHER - Interest	-0.17	0.33	-0.42
FRIEND - Provide Help	0.06	0.07	-0.19
FAMILY - Decision Making	0.02	0.21	0.11
TEACHER - Fairness	0.07	-0.01	0.01
LIFEWORTH	0.15	0.02	0.46
FAMILY - Provide Help	0.45	0.35	0.56
TEACHER - Trust	0.53	0.27	-0.03
FRIEND - Share Problems	0.50	0.67	0.40
FRIEND - Share Emo	0.63	0.34	0.71
FAMILY - Emo Support	1.22	1.12	0.88
FRIEND - Reliable Help	1.04	1.21	0.75
TEACHER - Care	1.21	1.32	1.80

Table S4. *Edge-weight results from the Network Comparison Test*

Var1	Var2	*p*-value	Test Statistic E
SUPPORT1	SUPPORT2	0.811881188	0.02278507
SUPPORT1	SUPPORT3	0.524752475	0.0612499
SUPPORT2	SUPPORT3	0.01980198	0.19288984
SUPPORT1	SUPPORT4	0.306930693	0.09570612
SUPPORT2	SUPPORT4	0.643564356	0.04331969
SUPPORT3	SUPPORT4	0.831683168	0.02234604
SUPPORT1	SUPPORT5	1	0
SUPPORT2	SUPPORT5	1	0
SUPPORT3	SUPPORT5	1	0
SUPPORT4	SUPPORT5	0.108910891	0.05912769
SUPPORT1	SUPPORT6	1	0
SUPPORT2	SUPPORT6	1	0
SUPPORT3	SUPPORT6	1	0
SUPPORT4	SUPPORT6	1	0
SUPPORT5	SUPPORT6	0.95049505	0.00529872
SUPPORT1	SUPPORT7	1	0
SUPPORT2	SUPPORT7	1	0
SUPPORT3	SUPPORT7	1	0
SUPPORT4	SUPPORT7	1	0
SUPPORT5	SUPPORT7	0.98019802	0.00325794
SUPPORT6	SUPPORT7	0.98019802	0.00085869
SUPPORT1	SUPPORT8	1	0
SUPPORT2	SUPPORT8	1	0
SUPPORT3	SUPPORT8	1	0
SUPPORT4	SUPPORT8	1	0
SUPPORT5	SUPPORT8	1	0.00082951
SUPPORT6	SUPPORT8	0.03960396	0.18530691
SUPPORT7	SUPPORT8	0.386138614	0.07624205
SUPPORT1	TEACHER1	1	0
SUPPORT2	TEACHER1	1	0
SUPPORT3	TEACHER1	1	0
SUPPORT4	TEACHER1	0.306930693	0.02790223
SUPPORT5	TEACHER1	1	0
SUPPORT6	TEACHER1	1	0
SUPPORT7	TEACHER1	1	0
SUPPORT8	TEACHER1	1	0
SUPPORT1	TEACHER2	1	0
SUPPORT2	TEACHER2	1	0
SUPPORT3	TEACHER2	1	0
SUPPORT4	TEACHER2	1	0
SUPPORT5	TEACHER2	1	0
SUPPORT6	TEACHER2	1	0
SUPPORT7	TEACHER2	1	0
SUPPORT8	TEACHER2	1	0
TEACHER1	TEACHER2	0.297029703	0.1272795
SUPPORT1	TEACHER3	1	0
SUPPORT2	TEACHER3	1	0
SUPPORT3	TEACHER3	1	0
SUPPORT4	TEACHER3	1	0

SUPPORT5	TEACHER3	1	0
SUPPORT6	TEACHER3	1	0
SUPPORT7	TEACHER3	1	0
SUPPORT8	TEACHER3	1	0
TEACHER1	TEACHER3	0.217821782	0.11624938
TEACHER2	TEACHER3	0.356435644	0.06705129
SUPPORT1	TEACHER5	1	0
SUPPORT2	TEACHER5	1	0
SUPPORT3	TEACHER5	1	0
SUPPORT4	TEACHER5	1	0
SUPPORT5	TEACHER5	0.02970297	0.02100678
SUPPORT6	TEACHER5	1	0
SUPPORT7	TEACHER5	1	0
SUPPORT8	TEACHER5	1	0
TEACHER1	TEACHER5	0.495049505	0.05303817
TEACHER2	TEACHER5	0.544554455	0.06180298
TEACHER3	TEACHER5	0.782178218	0.02847128
SUPPORT1	TEACHER6	1	0
SUPPORT2	TEACHER6	1	0
SUPPORT3	TEACHER6	1	0
SUPPORT4	TEACHER6	1	0
SUPPORT5	TEACHER6	1	0
SUPPORT6	TEACHER6	1	0
SUPPORT7	TEACHER6	1	0
SUPPORT8	TEACHER6	1	0
TEACHER1	TEACHER6	0.831683168	0.02091846
TEACHER2	TEACHER6	0.396039604	0.09766332
TEACHER3	TEACHER6	0.881188119	0.00859865
TEACHER5	TEACHER6	0.544554455	0.0599852
SUPPORT1	TEACHER8	1	0
SUPPORT2	TEACHER8	1	0
SUPPORT3	TEACHER8	1	0
SUPPORT4	TEACHER8	1	0
SUPPORT5	TEACHER8	1	0
SUPPORT6	TEACHER8	1	0
SUPPORT7	TEACHER8	1	0
SUPPORT8	TEACHER8	1	0
TEACHER1	TEACHER8	0.475247525	0.06483027
TEACHER2	TEACHER8	0.03960396	0.18256614
TEACHER3	TEACHER8	0.277227723	0.11646048
TEACHER5	TEACHER8	0.425742574	0.08127702
TEACHER6	TEACHER8	0.475247525	0.06017805
SUPPORT1	LIFESAT	1	0
SUPPORT2	LIFESAT	1	0
SUPPORT3	LIFESAT	1	0
SUPPORT4	LIFESAT	0.01980198	0.06529841
SUPPORT5	LIFESAT	1	0
SUPPORT6	LIFESAT	1	0
SUPPORT7	LIFESAT	1	0
SUPPORT8	LIFESAT	1	0
TEACHER1	LIFESAT	1	0
TEACHER2	LIFESAT	1	0
TEACHER3	LIFESAT	1	0

TEACHER5	LIFESAT	1	0
TEACHER6	LIFESAT	1	0
TEACHER8	LIFESAT	1	0
SUPPORT1	LIFEWORTH	1	0
SUPPORT2	LIFEWORTH	1	0
SUPPORT3	LIFEWORTH	0.03960396	0.03589035
SUPPORT4	LIFEWORTH	1	0
SUPPORT5	LIFEWORTH	1	0
SUPPORT6	LIFEWORTH	1	0
SUPPORT7	LIFEWORTH	1	0
SUPPORT8	LIFEWORTH	1	0
TEACHER1	LIFEWORTH	1	0
TEACHER2	LIFEWORTH	1	0
TEACHER3	LIFEWORTH	1	0
TEACHER5	LIFEWORTH	1	0
TEACHER6	LIFEWORTH	1	0
TEACHER8	LIFEWORTH	1	0
LIFESAT	LIFEWORTH	0.940594059	0.00371956
SUPPORT1	SYMPTOM4	1	0
SUPPORT2	SYMPTOM4	1	0
SUPPORT3	SYMPTOM4	1	0
SUPPORT4	SYMPTOM4	1	0
SUPPORT5	SYMPTOM4	1	0
SUPPORT6	SYMPTOM4	1	0
SUPPORT7	SYMPTOM4	1	0
SUPPORT8	SYMPTOM4	1	0
TEACHER1	SYMPTOM4	1	0
TEACHER2	SYMPTOM4	1	0
TEACHER3	SYMPTOM4	1	0
TEACHER5	SYMPTOM4	1	0
TEACHER6	SYMPTOM4	1	0
TEACHER8	SYMPTOM4	1	0
LIFESAT	SYMPTOM4	0.287128713	0.09239167
LIFEWORTH	SYMPTOM4	1	0
SUPPORT1	SYMPTOM5	1	0
SUPPORT2	SYMPTOM5	0.306930693	0.04723827
SUPPORT3	SYMPTOM5	0.198019802	0.10398952
SUPPORT4	SYMPTOM5	1	0
SUPPORT5	SYMPTOM5	1	0
SUPPORT6	SYMPTOM5	1	0
SUPPORT7	SYMPTOM5	1	0
SUPPORT8	SYMPTOM5	1	0
TEACHER1	SYMPTOM5	1	0
TEACHER2	SYMPTOM5	1	0
TEACHER3	SYMPTOM5	0.02970297	0.01707991
TEACHER5	SYMPTOM5	1	0
TEACHER6	SYMPTOM5	1	0
TEACHER8	SYMPTOM5	1	0
LIFESAT	SYMPTOM5	1	0
LIFEWORTH	SYMPTOM5	0.059405941	0.03891304
SYMPTOM4	SYMPTOM5	0.03960396	0.17058626
SUPPORT1	SYMPTOM6	1	0
SUPPORT2	SYMPTOM6	1	0

SUPPORT3	SYMPTOM6	1	0
SUPPORT4	SYMPTOM6	1	0
SUPPORT5	SYMPTOM6	1	0
SUPPORT6	SYMPTOM6	1	0
SUPPORT7	SYMPTOM6	1	0
SUPPORT8	SYMPTOM6	1	0
TEACHER1	SYMPTOM6	1	0
TEACHER2	SYMPTOM6	1	0
TEACHER3	SYMPTOM6	1	0
TEACHER5	SYMPTOM6	1	0
TEACHER6	SYMPTOM6	1	0
TEACHER8	SYMPTOM6	1	0
LIFESAT	SYMPTOM6	1	0
LIFEWORTH	SYMPTOM6	1	0
SYMPTOM4	SYMPTOM6	0.801980198	0.02299847
SYMPTOM5	SYMPTOM6	0.544554455	0.05116594
SUPPORT1	SYMPTOM7	1	0
SUPPORT2	SYMPTOM7	0.099009901	0.0159345
SUPPORT3	SYMPTOM7	1	0
SUPPORT4	SYMPTOM7	0.198019802	0.02704423
SUPPORT5	SYMPTOM7	1	0
SUPPORT6	SYMPTOM7	1	0
SUPPORT7	SYMPTOM7	1	0
SUPPORT8	SYMPTOM7	1	0
TEACHER1	SYMPTOM7	0.01980198	0.0335485
TEACHER2	SYMPTOM7	1	0
TEACHER3	SYMPTOM7	1	0
TEACHER5	SYMPTOM7	1	0
TEACHER6	SYMPTOM7	1	0
TEACHER8	SYMPTOM7	1	0
LIFESAT	SYMPTOM7	0.792079208	0.01376278
LIFEWORTH	SYMPTOM7	1	0
SYMPTOM4	SYMPTOM7	0.435643564	0.05997644
SYMPTOM5	SYMPTOM7	0.445544554	0.06903391
SYMPTOM6	SYMPTOM7	0.712871287	0.04027355
SUPPORT1	MNTLHLTH	1	0
SUPPORT2	MNTLHLTH	1	0
SUPPORT3	MNTLHLTH	1	0
SUPPORT4	MNTLHLTH	1	0
SUPPORT5	MNTLHLTH	1	0
SUPPORT6	MNTLHLTH	1	0
SUPPORT7	MNTLHLTH	1	0
SUPPORT8	MNTLHLTH	1	0
TEACHER1	MNTLHLTH	1	0
TEACHER2	MNTLHLTH	1	0
TEACHER3	MNTLHLTH	1	0
TEACHER5	MNTLHLTH	1	0
TEACHER6	MNTLHLTH	1	0
TEACHER8	MNTLHLTH	1	0
LIFESAT	MNTLHLTH	0.277227723	0.10032049
LIFEWORTH	MNTLHLTH	1	0
SYMPTOM4	MNTLHLTH	0.910891089	0.00655484
SYMPTOM5	MNTLHLTH	1	0

SYMPTOM6	MNTLHLTH	0.584158416	0.04087566
SYMPTOM7	MNTLHLTH	0.336633663	0.03053205
SUPPORT1	WELLOFF	0.158415842	0.07301196
SUPPORT2	WELLOFF	0.386138614	0.04410034
SUPPORT3	WELLOFF	1	0
SUPPORT4	WELLOFF	1	0
SUPPORT5	WELLOFF	1	0
SUPPORT6	WELLOFF	1	0
SUPPORT7	WELLOFF	1	0
SUPPORT8	WELLOFF	1	0
TEACHER1	WELLOFF	1	0
TEACHER2	WELLOFF	1	0
TEACHER3	WELLOFF	1	0
TEACHER5	WELLOFF	1	0
TEACHER6	WELLOFF	1	0
TEACHER8	WELLOFF	1	0
LIFESAT	WELLOFF	0.514851485	0.0174153
LIFEWORTH	WELLOFF	1	0
SYMPTOM4	WELLOFF	1	0
SYMPTOM5	WELLOFF	1	0
SYMPTOM6	WELLOFF	1	0
SYMPTOM7	WELLOFF	1	0
MNTLHLTH	WELLOFF	1	0
SUPPORT1	SAFETY	1	0
SUPPORT2	SAFETY	1	0
SUPPORT3	SAFETY	1	0
SUPPORT4	SAFETY	1	0
SUPPORT5	SAFETY	1	0
SUPPORT6	SAFETY	1	0
SUPPORT7	SAFETY	1	0
SUPPORT8	SAFETY	1	0
TEACHER1	SAFETY	1	0
TEACHER2	SAFETY	1	0
TEACHER3	SAFETY	1	0
TEACHER5	SAFETY	1	0
TEACHER6	SAFETY	1	0
TEACHER8	SAFETY	1	0
LIFESAT	SAFETY	0.297029703	0.01946983
LIFEWORTH	SAFETY	1	0
SYMPTOM4	SAFETY	1	0
SYMPTOM5	SAFETY	1	0
SYMPTOM6	SAFETY	1	0
SYMPTOM7	SAFETY	1	0
MNTLHLTH	SAFETY	1	0
WELLOFF	SAFETY	1	0
SUPPORT1	DISCRIM3	0.04950495	0.09046357
SUPPORT2	DISCRIM3	1	0
SUPPORT3	DISCRIM3	1	0
SUPPORT4	DISCRIM3	1	0
SUPPORT5	DISCRIM3	1	0
SUPPORT6	DISCRIM3	1	0
SUPPORT7	DISCRIM3	1	0
SUPPORT8	DISCRIM3	1	0

TEACHER1	DISCRIM3	1	0
TEACHER2	DISCRIM3	0.00990099	0.16393242
TEACHER3	DISCRIM3	1	0
TEACHER5	DISCRIM3	1	0
TEACHER6	DISCRIM3	1	0
TEACHER8	DISCRIM3	1	0
LIFESAT	DISCRIM3	1	0
LIFEWORTH	DISCRIM3	1	0
SYMPTOM4	DISCRIM3	1	0
SYMPTOM5	DISCRIM3	0.01980198	0.40415124
SYMPTOM6	DISCRIM3	1	0
SYMPTOM7	DISCRIM3	1	0
MNTLHLTH	DISCRIM3	1	0
WELLOFF	DISCRIM3	1	0
SAFETY	DISCRIM3	0.02970297	0.02897135
SUPPORT1	PHYSHLTH	1	0
SUPPORT2	PHYSHLTH	1	0
SUPPORT3	PHYSHLTH	1	0
SUPPORT4	PHYSHLTH	1	0
SUPPORT5	PHYSHLTH	1	0
SUPPORT6	PHYSHLTH	1	0
SUPPORT7	PHYSHLTH	1	0
SUPPORT8	PHYSHLTH	1	0
TEACHER1	PHYSHLTH	1	0
TEACHER2	PHYSHLTH	1	0
TEACHER3	PHYSHLTH	1	0
TEACHER5	PHYSHLTH	1	0
TEACHER6	PHYSHLTH	0.118811881	0.01513418
TEACHER8	PHYSHLTH	1	0
LIFESAT	PHYSHLTH	0.168316832	0.1111183
LIFEWORTH	PHYSHLTH	0.237623762	0.0573136
SYMPTOM4	PHYSHLTH	1	0
SYMPTOM5	PHYSHLTH	1	0
SYMPTOM6	PHYSHLTH	1	0
SYMPTOM7	PHYSHLTH	1	0
MNTLHLTH	PHYSHLTH	0.920792079	0.00407152
WELLOFF	PHYSHLTH	0.772277228	0.02461515
SAFETY	PHYSHLTH	0.207920792	0.05721181
DISCRIM3	PHYSHLTH	1	0
SUPPORT1	BELONGING	1	0
SUPPORT2	BELONGING	1	0
SUPPORT3	BELONGING	1	0
SUPPORT4	BELONGING	1	0
SUPPORT5	BELONGING	1	0
SUPPORT6	BELONGING	0.574257426	0.01328121
SUPPORT7	BELONGING	1	0
SUPPORT8	BELONGING	1	0
TEACHER1	BELONGING	1	0
TEACHER2	BELONGING	0.257425743	0.04113973
TEACHER3	BELONGING	1	0
TEACHER5	BELONGING	0.188118812	0.04570257
TEACHER6	BELONGING	1	0
TEACHER8	BELONGING	1	0

LIFESAT	BELONGING	0.475247525	0.02028324
LIFEWORTH	BELONGING	0.653465347	0.04323335
SYMPTOM4	BELONGING	1	0
SYMPTOM5	BELONGING	1	0
SYMPTOM6	BELONGING	0.346534653	0.03817303
SYMPTOM7	BELONGING	1	0
MNTLHLTH	BELONGING	1	0
WELLOFF	BELONGING	1	0
SAFETY	BELONGING	1	0
DISCRIM3	BELONGING	1	0
PHYSHLTH	BELONGING	0.534653465	0.05306531
SUPPORT1	DAILYSTRESS_R	1	0
SUPPORT2	DAILYSTRESS_R	0.03960396	0.03937597
SUPPORT3	DAILYSTRESS_R	1	0
SUPPORT4	DAILYSTRESS_R	1	0
SUPPORT5	DAILYSTRESS_R	1	0
SUPPORT6	DAILYSTRESS_R	1	0
SUPPORT7	DAILYSTRESS_R	1	0
SUPPORT8	DAILYSTRESS_R	1	0
TEACHER1	DAILYSTRESS_R	1	0
TEACHER2	DAILYSTRESS_R	1	0
TEACHER3	DAILYSTRESS_R	1	0
TEACHER5	DAILYSTRESS_R	1	0
TEACHER6	DAILYSTRESS_R	1	0
TEACHER8	DAILYSTRESS_R	1	0
LIFESAT	DAILYSTRESS_R	0.623762376	0.04363235
LIFEWORTH	DAILYSTRESS_R	1	0
SYMPTOM4	DAILYSTRESS_R	0.98019802	0.00213479
SYMPTOM5	DAILYSTRESS_R	1	0
SYMPTOM6	DAILYSTRESS_R	0.673267327	0.03616671
SYMPTOM7	DAILYSTRESS_R	0.683168317	0.03638702
MNTLHLTH	DAILYSTRESS_R	0.762376238	0.03521005
WELLOFF	DAILYSTRESS_R	1	0
SAFETY	DAILYSTRESS_R	1	0
DISCRIM3	DAILYSTRESS_R	1	0
PHYSHLTH	DAILYSTRESS_R	1	0
BELONGING	DAILYSTRESS_R	1	0
